# Preliminary Evaluation of a Web-Based International Journal Club for Ketamine in Psychiatric Disorders: Cross-Sectional Survey Study

**DOI:** 10.2196/46158

**Published:** 2023-11-01

**Authors:** Jacek R Lindner, Ashkan Ebrahimi, Julian F Kochanowicz, Justyna Szczupak, Timothy Paris, Ahmed Abdelsamie, Sagar V Parikh, Rupert McShane, Sara Costi

**Affiliations:** 1 Interventional Psychiatry Service Warneford Hospital Oxford Health NHS Foundation Trust Oxford United Kingdom; 2 Medicine Program Poznan University of Medical Sciences Poznan Poland; 3 Department of Urology Vivantes Auguste-Viktoria-Klinikum Berlin Germany; 4 South London and Maudsley NHS Foundation Trust London United Kingdom; 5 Warneford Hospital Oxford Health NHS Foundation Trust Oxford United Kingdom; 6 King's College London London United Kingdom; 7 Department of Psychiatry University of Michigan Ann Arbor, MI United States; 8 Department of Psychiatry University of Oxford Oxford United Kingdom; 9 Department of Psychiatry Icahn School of Medicine at Mount Sinai New York, NY United States

**Keywords:** web-based journal club, journal club, remote learning, ketamine, medical education, web-based, survey, COVID-19, psychiatry, evaluation, YouTube, networking, internet, format

## Abstract

**Background:**

The use of novel rapid-acting antidepressants for psychiatric disorders is expanding. The web-based Ketamine and Related Compounds International Journal Club (KIJC) was created during the COVID-19 pandemic by UK academic psychiatrists and trainees for interested global professionals to discuss papers related to the topic of ketamine for the treatment of psychiatric disorders. The KIJC aimed to facilitate bidirectional discussions, sharing of ideas, and networking among participants.

**Objective:**

The aim of this study is a preliminary evaluation of the journal club’s format for satisfaction and impact after the first year of running.

**Methods:**

A website, email, and word of mouth were used for recruitment. The journal club was held twice per month using videoconferencing software in 3 parts: a 20-minute presentation, a 15-minute chaired question and answer session, and a 25-minute informal discussion with participants’ cameras on. The first 2 parts were recorded and uploaded to the website alongside links to the corresponding papers. In total, 24 speakers presented from 8 countries, typically within 2 (SD 2) months of publication. The average attendance was 51 (SD 20) audience members, and there were 63 (SD 50) views of each subsequent recording. Two anonymous web-based cross-sectional surveys were conducted from November 2021 to February 2022, one for speakers and another for audience members, separately. Various survey statements, 14 for speakers and 12 for the audience, were categorized according to satisfaction and impact, alongside obtaining participants’ primary career roles and requesting optional written feedback. Responses were compared between both groups and analyzed, including an inductive thematic analysis and a summary of lessons learned.

**Results:**

A total of 30 survey responses were obtained, demonstrating overall agreement with the statements. In total, 12 (50%) out of 24 speakers and 18 (35%) out of an average of 51 (SD 20) audience members regarded the journal club’s format as satisfying and impactful. The majority (26/30, 87%) of respondents identified as clinicians (9/30, 30%), researchers (9/30, 30%), and clinician-researchers (8/30, 27%). Additionally, 11 (37%) of the 30 respondents also provided optional written feedback: 3 (10%) speakers and 8 (27%) audience members. From the written feedback, 5 main themes were derived: engagement with the journal club, desire for active participation, improving the platform, positive learning experiences, and suggestions for future sessions.

**Conclusions:**

The journal club successfully reached its intended audience and developed into a web-based community. The majority of the participants were satisfied with the format and found it impactful. Overall, the journal club appears to be a valuable tool for knowledge sharing and community building in the field of ketamine use for the treatment of psychiatric disorders. A larger sample size and additional testing methods are required to support the generalizability of the journal club’s format.

## Introduction

In the wake of the COVID-19 pandemic, a group of academic clinicians at the University of Oxford and psychiatry trainees in the United Kingdom recognized the need for a platform to share knowledge about ketamine and related compounds in psychiatry. Their ultimate goal was to connect researchers and clinical practitioners. This initiative led to the establishment of the Ketamine and Related Compounds International Journal Club (KIJC), subsequently referred to as the journal club.

When designing the journal club, the group took into consideration the challenges documented in a 2020 publication about running journal clubs [1]. They also responded to the demand for innovative approaches to medical education during the pandemic [2], offering a fresh way to keep up-to-date with the rapidly evolving field of ketamine in psychiatry. Furthermore, existing evidence [3] suggested the advantages of web-based conferences, such as increased accessibility and enhanced interactions. A recent study [4] also demonstrated the effectiveness of videoconferencing technology in delivering training courses. Additionally, a paper authored by Raby et al [5] highlighted the benefits of hosting web-based academic conferences for free and addressed potential barriers to delegate participation that needed to be overcome.

Drawing inspiration from these sources, the journal club was designed as a voluntary extracurricular web-based platform, offered at no cost. It targeted an audience comprising clinical and academic professionals with bimonthly meetings to discuss recently published papers related to ketamine and related compounds in psychiatry. The journal club aimed to facilitate 2-way discussions, idea sharing, and networking among participants.

The purpose of this paper is to discuss the design, implementation, and initial evaluation of the innovative format of the KIJC.

## Methods

### Overview

Based on the findings of a pilot study [3], which validated the effectiveness of a synchronous web-based journal club with a similar format, a free web-based meeting was held twice per month for the first year. Zoom (Zoom Video Communications, Inc) was chosen as the hosting platform, using the webinar function with an emailed link. A website, email, and word of mouth were used for recruitment. The webinars were conducted in English, ensuring consistency, and were scheduled to accommodate participants from different time zones. Each webinar had a duration of 1 hour. The scheduling recommendations, as per best practices [6], were considered with an emphasis on regularity. The timing aimed to accommodate the breakfast and lunchtime hour for a speculated majority audience from the Western and Eastern United States, respectively. This corresponded to the evening time for the European audiences. The format was structured into 3 distinct parts. Part 1 involved a live presentation delivered by a visible speaker, who was always 1 of the paper’s authors, for approximately 20 minutes. Part 2 featured a recorded 15-minute chaired question and answer (Q&A) session, where the nonvisible live audience submitted questions via the Q&A function. Part 3 consisted of an unrecorded synchronous continuation of the journal club, where all attendees were promoted to panelists, allowing for a live-only 25-minute informal discussion with everyone’s cameras and microphones turned on. Each session concluded after 1 hour, facilitated by one of the hosts. The recorded first 2 parts were made available indefinitely for free viewing on the journal club’s website and on the journal club’s YouTube (Google LLC) channel. This provided outreach to broader audiences [7], and it also added web-based educational videos for the general public and for health care providers [8]. Papers for the presentation were chosen by senior academic clinicians through PubMed searches using relevant keywords related to ketamine, mental health conditions, and psychotherapy. The speakers were then contacted via email, and once confirmed, invitations were sent to audience members on the mailing list via email, along with links to past research papers and recordings, enabling both attendees and speakers to prepare for presentations. Further information was also available through the journal club website [9].

During the first year, the web-based journal club met 24 times. The papers presented encompassed a variety of study types, including randomized controlled trials (n=11), preclinical studies (n=3), experimental medicine and human mechanistic studies (n=2), case series (n=1), retrospective analyses (n=5), and systematic reviews (n=2). The presentations by the speaking authors (n=24) occurred, on average, within 2 (SD 2) months of the publication of their respective papers, with 12 (50%) speakers taking place within 1 month of publication.

Each journal club presentation attracted an average number of 51 (SD 20) live participants and an average number of 63 (SD 50) viewings of the recordings (n=24). Approximately half (26/51, 51%) of the average live participants constituted a consistent group, with the majority (22/26, 85%) being clinicians. Audience members were required to preregister for each Zoom webinar individually via emailed links. Upon logging into each webinar, attendees were greeted by recurring hosts who explained the format, emphasized the distinction between the recorded and unrecorded parts, and introduced the speakers. During the first 2 live recorded parts, attendees were not visible and muted, while the hosts and speaker were visible and heard and listed as panelists. Attendees could submit questions via the Q&A or chat functions, which were read out loud by the chairing hosts during the allocated time. The remaining questions were encouraged to be asked in person during the informal discussions with the presenting author. After the speaker’s presentation, all attendees were upgraded to panelists, allowing them to turn on their cameras and microphones too, for an unmoderated and less formal discussion.

After the first year, a preliminary evaluation was completed on the journal club’s novel design and format. The 2 anonymous web-based cross-sectional surveys were conducted using the web tool SurveyMonkey (SurveyMonkey Inc) from November 2021 to February 2022, one for speakers and another for audience members, separately. Varying survey statements, 14 for speakers and 12 for the audience, were categorized according to format satisfaction, format impact, obtaining participants’ primary career roles, and requesting optional written feedback.

Past speakers received personalized email invitations to participate in the speaker survey (Multimedia Appendix 1). The speaker survey consisted of 14 statements for respondents to agree, neither agree nor disagree, or disagree with. Respondents were also asked to indicate their primary career roles and had the option to provide further written comments. The audience survey was conducted simultaneously with the speaker survey using the same web tool (Multimedia Appendix 2). The general invitation link for the audience survey was included in the webinar invitations sent to all recipients registered on the journal club mailing list, regardless of attendance. Reminders to complete the surveys were provided during live events. Similar to the speaker survey, respondents were presented with 12 statements to rank their agreement, neutrality, or disagreement. Primary career roles were also collected, and respondents had the opportunity to provide additional written comments.

The survey design was inspired by classification metrics used by clinicians in the United States for evaluating web-based medical education in psychiatry [4]. Survey responses were compared between both groups and summarized along with the main lessons learned.

### Statistical Analysis

All statistical analysis was performed using JASP (The JASP Team) statistical software. Fisher exact test was conducted to determine the statistical significance of the differences in agreement between the 2 groups surveyed, the speakers and the audience, for statements that had a discernible difference in opinion. Determination of significance was done using a *P* value threshold of .05. For the qualitative results, a thematic analysis of the survey written feedback was performed using an inductive approach by 2 of the study authors who derived themes from the data, and relevant explanations were summarized.

### Ethical Considerations

The ethical considerations for this project were in line with the guidelines of the UK National Health Service (NHS). The project was deemed as an evaluation of a web-based journal club and not classified as research requiring review by an NHS Research Ethics Committee or the NHS Research and Development Office. Informed consent was obtained from participants before they participated in the surveys, and all responses were anonymous. The UK Government research exemption allowed access to papers for personal study purposes. Participants were informed about the recording of journal club presentations.

## Results

**Figure 1:**
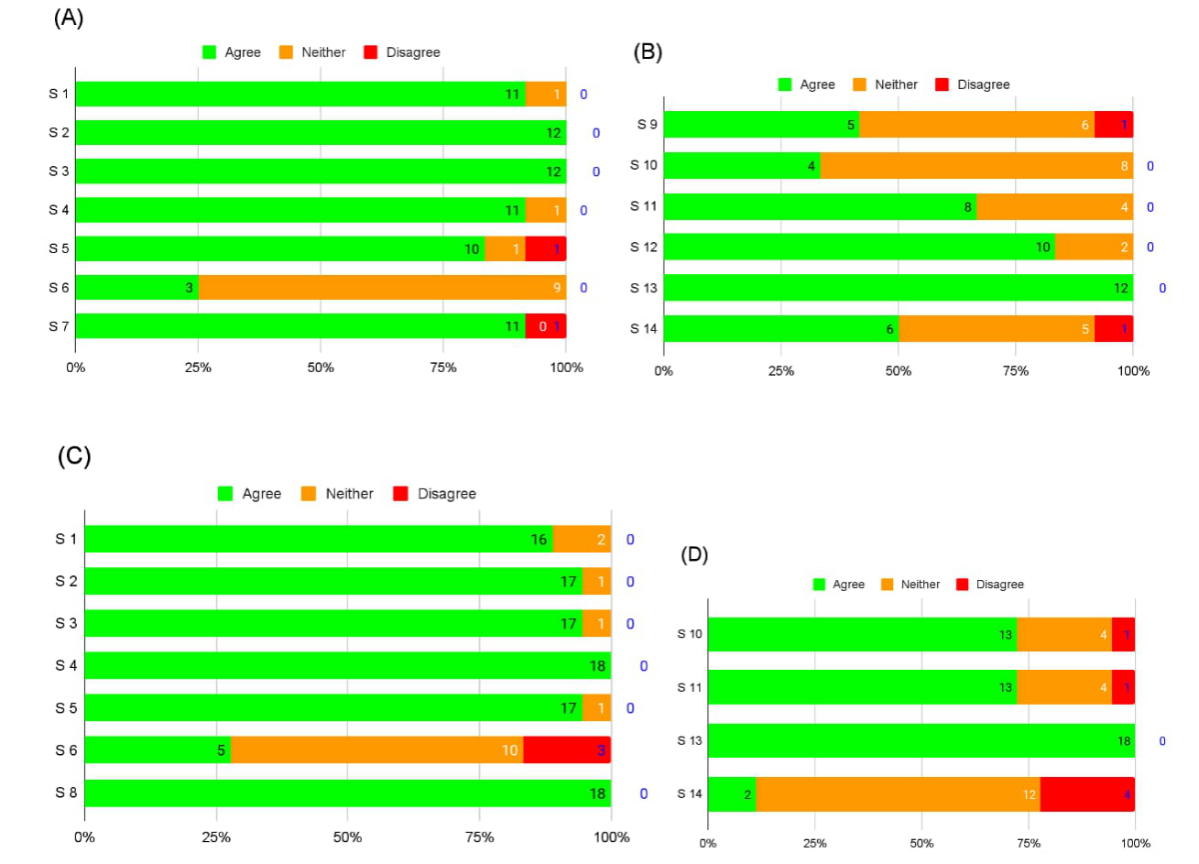
Both speaker and audience survey responses to the satisfaction- and impact-related statements (S1-S14), respectively (see Table 1 for complete descriptions of each statement), on a 3-point Likert scale (green: agree, orange: neither agree nor disagree, and red: disagree). (A) Speaker satisfaction (B) Speaker impact (C) Audience satisfaction (D) Audience impact.

The data collected from the speakers' and audience members' survey responses are presented in Table 1. The entire survey sample across all groups studied included responses from a total of 30 participants, with 12 (50%) out of 24 speakers and 18 (35%) out of an average of 51 (SD 20) audience members participating. In the speakers’ survey (n=12), 6 (50%) were clinician-researchers, 5 (42%) were researchers, and 1 (8%) was a student. Responders within the audience survey (n=18) included 9 (50%) clinicians, 4 (22%) researchers, 2 (11%) clinician-researchers, 2 (11%) students, and 1 (6%) therapist. A graphical representation of the results from the speaker and audience surveys is shown in Figure 1.

**Table 1:** Survey results from the speakers (n=12) and audience (n=18) and statistical analysis using the Fisher exact test for both speaker and audience survey responses to the satisfaction and impact-related statements.

Statement		Agree, n (%)^a^			Neither agree nor disagree, n (%)			Disagree, n (%)			Fisher exact test, *P* value^b^
		S^c^ (n=12)	A^d^ (n=18)	S (n=12)		A (n=18)	S (n=12)		A (n=18)		
**Format satisfaction**											
	(1) Journal club follows a novel format of web-based presenting	11 (92)	16 (89)	1 (8)		2 (11)	0 (0)		0 (0)	≥.99	
	(2) The format is engaging for both the speaker and the audience	12 (100)	17 (94)	0 (0)		1 (6)	0 (0)		0 (0)	≥.99	
	(3) I am satisfied with the 20 minutes time frame for the speaker presentations	12 (100)	17 (94)	0 (0)		1 (6)	0 (0)		0 (0)	≥.99	
	(4) I am satisfied with the 15 minutes time frame for the chaired Q&A^e^ session	11 (92)	18 (100)	1 (8)		0 (0)	0 (0)		0 (0)	.40	
	(5) I am satisfied with the 25 minutes time frame for the informal discussion with the attendees	10 (83)	17 (94)	1 (9)		1 (6)	1 (8)		0 (0)	.50	
	(6) I prefer the informal discussion with the attendees over the chaired Q&A session	3 (25)	5 (28)	9 (75)		10 (55)	0 (0)		3 (17)	.30	
	(7) I had enough time in advance to prepare for my presentation	11 (92)	N/A^f^	0 (0)		N/A	1 (8)		N/A	N/A	
	(8) I am satisfied with the quality of the speakers and their presentations	N/A	18 (100)	N/A		0 (0)	N/A		0 (0)	N/A	
**Format impact**											
	(9) I would modify other presentations to mimic the novel journal club presenting format	5 (42)	N/A	6 (50)		N/A	1 (8)		N/A	N/A	
	(10) I believe that the informal discussion with the attendees may influence my clinical practice	4 (33)	13 (72)	8 (67)		4 (22)	0 (0)		1 (6)	.02	
	(11) I believe that the informal discussion with the attendees may influence my research	8 (67)	13 (72)	4 (33)		4 (22)	0 (0)		1 (6)	.81	
	(12) I would recommend this format of presenting to others	10 (83)	N/A	2 (17)		N/A	0 (0)		N/A	N/A	
	(13) I would recommend journal club to others	12 (100)	18 (100)	0 (0)		0 (0)	0 (0)		0 (0)	N/A	
	(14) I developed new contacts from the informal discussion with the attendees	6 (50)	2 (11)	5 (42)		12 (67)	1 (8)		4 (22)	.08	

^a^Percentages were rounded off to the nearest whole number.

^b^*P* value threshold was set at .05.

^c^S: speakers.

^d^A: audience.

^e^Q&A: question and answer.

^f^N/A: not applicable.

Fisher exact testing was used to detect any nonrandom associations between the agreement percentages for satisfaction and impact-related statements from speakers and the audience in Table 1. Differing statements (7, 8, 9, and 12) between the 2 surveys and an unanimously agreed statement (13) in both surveys were excluded. Regarding satisfaction-related statements, no significant differences in agreement between speakers and the audience emerged. Statements 1, 2, 3, 4, 5, and 6 did not show any statistically significant differences between groups (*P*>.05). Of note, within impact-related statements, statement 10 showed a significant difference in agreement (*P*=.02; *P*<.05), suggesting varied perceptions of impact. However, statements 11 and 14 did not exhibit significant differences (*P*>.05).

Furthermore, 11 (37%) respondents also provided anonymous optional written feedback (Multimedia Appendix 3) individually from 3 (10%) speakers and 8 (27%) audience members. An inductive thematic analysis was performed by 2 researchers who analyzed and coded the responses. The analysis produced five main themes: (1) Engagement with the journal club: both presenters and attendees expressed delight in attending the journal club and connecting with experts and other participants. They noted that the journal club had developed into a strong community. Some speakers indicated their willingness to be more actively involved. (2) Desire for active participation: audience members expressed a desire for a more active role. For example, they suggested enabling audience cameras during sessions and allowing time for more questions, aiming to enhance interaction. (3) Improving the platform: some audience members noticed issues with receiving notifications and invites. They also requested that the sessions be recorded and made available for later review. (4) Positive learning experiences: both presenters and the audience noted that they derived value from the journal club. They praised the balance between clinical and basic science and the quality of both presenters and attendees. (5) Suggestions for future sessions: some expressed interest in hearing from specific speakers on topics such as dextromethorphan, nitrous oxide, ketamine, and rapamycin. They also wished to hear from the organizers about their experience working in a ketamine clinic.

## Discussion

### Principal Findings

First, the speakers and audience members surveyed were largely satisfied in terms of statements (1-8) pertaining to the journal club's format and delivery. Regarding statements (9-14) relating to the impact of the journal club, both the audience and speaking authors overall agreed on the impact the journal club had on their clinical practice or further research, and all those surveyed collectively agreed (statement 13) they would recommend the journal club to colleagues.

Second, the journal club aimed to reach an audience likely familiar with the general background of ketamine treatment for psychiatric disorders and evolved into a web-based community comprising clinicians, clinician-researchers, basic researchers, therapists, and students. Overall, the journal club combined elements of a synchronous journal club [10] and a “digital platform” approach [11] by providing links to publications for personal study ahead of time and creating a digital library for free on-demand viewing of past recordings. As suggested by Stefanoudis et al [12], wherein participants of a web-based German conference expressed their willingness to have the contents of the sessions permanently available for later viewing [13], this allowed interested individuals to explore and watch the KIJC sessions at a later time.

Third, there was 92% (11/12) agreement to statement 7, “I had enough time in advance to prepare for my presentation,” by the responding speakers and 100% (18/18) agreement to statement 8, “I am satisfied with the quality of the speakers and their presentations,” by all audience members surveyed. The quick turnaround time between the publication and presentation of research papers at the journal club (mean 2, SD 2 mo; 12/24, 50% speakers taking place within 1 month of publication) is an advantage for both the audience and the presenting researchers who have the material at their fingertips and so the burden of presenting is reduced.

Fourth, statement 10 (*P*=.02; *P*<.05) produced the only statistically significant nonrandom association in opinion between both surveys, “I believe that the informal discussion with the attendees may influence my clinical practice.” Influencing clinical practice is a timely process with clinicians needing time to deem the appropriate changes necessary and implement modifications to their own practice. This statement’s relevance also mainly targets clinicians, and it is possible those who voted neither to agree nor disagree were not involved in clinical practice and may have wanted to abstain or remain neutral if this did not apply to them.

Fifth, the opportunity for brainstorming among the participants was expected mostly from the unrecorded informal discussions during part 3 of the webinars where participants were encouraged to turn on their cameras and microphones. As per statement 11, “I believe that the informal discussion with the attendees may influence my research,” this aspect was rated highly not only by the audience but perhaps more importantly by the speakers. The journal club being useful to the researchers too is overlooked and different from a conventional journal club which does not involve the authors. The free flow of conversation allowed the researchers to understand the relevance of their work to a predominantly clinical audience. Some researchers particularly valued the interaction as it helped them to see where clinical priorities lie and to understand more directly how their work was received by potential users. This is pretty unique as researchers in conferences rarely have prolonged discussions, and in specialist presentations, the voice of the end user tends to be quieter than that of researchers working in the same field. It is a feature that arises because of this particular 3-part format of the journal club.

Sixth, there were mixed reviews for statement 14, “I developed new contacts from the informal discussion with the attendees,” for both speakers and audiences, possibly due to the journal club format. As shown before, group discussions enhance audience engagement and satisfaction, which are deemed common problems of web-based meetings and webinars [14]. Networking and developing new contacts from web-based sessions, especially for individuals who joined for the first time, can be a challenging process. During the informal unrecorded discussion, only individuals who are willing to actively participate would have a higher likelihood of having conversations with other participants. Alternatively, it is possible that the journal club format and wider web-based attendance at journal clubs are not as conducive to networking as the group believed it to be. A recent study [5] highlighted the difficulties in replicating spontaneous human encounters during web-based academic events in comparison to those hosted in person. A potential change could be holding an annual in-person conference and inviting all the web-based members to attend to encourage meeting one another and then re-evaluating this item after subsequent web-based encounters. It is likely that other solutions are needed to promote networking in the web-based journal club community.

Finally, from the group’s collective experience of organizing the web-based KIJC and reviewing the 2 cross-sectional survey responses after 1 year, the main lessons learned were summarized as follows: (1) a consistent and routine schedule was maintained for webinars to accommodate a mixed audience of participants from varying time zones, (2) clear instructions and explanations of the format of each journal club meeting were provided at the beginning of each session, (3) encouraging attendees to turn on cameras and microphones during the informal discussion to foster engagement and dialog among attendees, (4) adapting and adjusting the format continuously to address the changing requirements and interests of the audience, and (5) publishing recorded segments on the journal club website and YouTube channel to expand the outreach of the journal club and to allow access to those who were not able to attend live sessions.

### Limitations

There are several limitations to this work. First, the limited response rate to the audience and speaker surveys limits the generalizability of the findings. It is likely that survey respondents were more likely to rate the journal club favorably than nonrespondents. An average number of 51 (SD 20) live participants and an average number of 63 (SD 50) views of the recordings indicate a substantial degree of variability in the club’s attendance. The number of attendees varied depending on perhaps both the speaker and topics presented, with different speakers and topics drawing different audience sizes, and thus, the delivery of live and recorded invitations to the surveys varied. To increase future participation in surveys, issuing a journal club participation certificate could be considered, as it resulted in a 100% response rate by a recent student-run, web-based journal club initiative in Turkey [15].

Second, the general invitation link for the survey was included in the webinar invitations sent to all recipients registered on the journal club mailing list, regardless of attendance, and no measures were in place to guarantee that only attendees were responding to the survey or that speakers were not also answering audience surveys.

Third, the survey did not assess the participants’ satisfaction with the frequency of the journal club’s webinars. A study showed that physicians felt overwhelmed and struggled to participate in the extensive offer of webinars during the COVID-19 pandemic [16].

Finally, the degree to which the journal club influences the clinical and research practice of participants remains subjective and based solely on self-report. A recent group [17] of educators highlighted the importance of evaluation tools and suggested the completion of preintervention and postintervention surveys to assess participant knowledge and self-perceived competence prior to and after the use of a web-based tutorial, which could have been implemented. The journal club and other groups involved in similar initiatives should consider methods for evaluating the long-term behavioral outcomes from web-based journal club attendance, such as knowledge testing. This could also be compared with the use of other educational interventions, from more conventional in-person events to hybrid and web-based conferences.

### Conclusions

The journal club successfully reached its intended audience and developed into a web-based community. The majority of the participants were satisfied with the format and found it impactful. Overall, the web-based journal club is a valuable tool for knowledge sharing and community building in the field of ketamine use for the treatment of psychiatric disorders. A larger sample size and further testing methods are needed to support the generalizability of the web-based journal club’s format.

## Appendix

Multimedia Appendix 1 Ketamine international journal club: speaker survey.

Multimedia Appendix 2 Ketamine international journal club: audience survey.

Multimedia Appendix 3 Ketamine international journal club: survey written feedback.

## Abbreviations

KIJC: Ketamine and Related Compounds International Journal Club
NHS: National Health Service
Q&A: question and answer

## Data Availability

All survey data generated or analyzed during this study are included in this published paper and Multimedia Appendices 1-3.
