# Supplementary material for: Preliminary Evaluation of a Web-Based International Journal Club for Ketamine in Psychiatric Disorders: Cross-Sectional Survey Study
Source: JMIR Med Educ. 2023 Nov 1;9:e46158. doi: 10.2196/46158 (PMC10652200; doi:10.2196/46158)
Supplement: Multimedia Appendix 1 [file mededu_v9i1e46158_app1.pdf]

Ketamine International Journal Club: Speaker Feedback

1-minute survey

Please select the degree to which you agree with the following statements.

OK

* 1. This is a novel format of online presenting (20min presentation/ 15min chaired Q&A/ 25min infor
discussion with attendees 'cameras on').

- Agree
- Neither agree nor disagree
- Disagree

* 2. This format of presenting is engaging for the speaker and the audience.

- Agree
- Neither agree nor disagree
- Disagree

* 3. I will modify other presentations to mimic this format.

- Agree
- Neither agree nor disagree
- Disagree

* 4. I would recommend this format of presenting to others.

- Agree
- Neither agree nor disagree
- Disagree

0 of 14 answered

* 5. I am satisfied with the time for speaker presentations (20 minutes).

- Agree
- Neither agree nor disagree
- Disagree

* 6. I am satisfied with the time for the chaired Q&A sessions (15 minutes).

- Agree
- Neither agree nor disagree
- Disagree

* 7. I am satisfied with the time for informal discussion with attendees (25 minutes).

- Agree
- Neither agree nor disagree
- Disagree

* 8. I prefer the informal discussion with attendees more than the chaired Q&A sessions.

- Agree
- Neither agree nor disagree
- Disagree

* 9. The informal discussion with attendees may influence my clinical practice.

- Agree
- Neither agree nor disagree
- Disagree

* 10. The informal discussion with attendees may influence my research.

- Agree
- Neither agree nor disagree
- Disagree

* 11. I have developed new contacts from the informal discussion with attendees.

- Agree
- Neither agree nor disagree
- Disagree

* 12. I had enough time in advance to prepare for my presentation.

- Agree
- Neither agree nor disagree
- Disagree

* 13. Please indicate your primary role:

- Clinician
- Student
- Researcher
- Psychologist
- Clinician-Researcher
- Therapist
- Other (please specify)

* 14. I would recommend this journal club.

- Agree
- Neither agree nor disagree
- Disagree

Please provide any further feedback below (i.e. what could we do differently?):

DONE

Powered by
SurveyMonkey
See how easy it is to create a survey.
