# Supplementary material for: Preliminary Evaluation of a Web-Based International Journal Club for Ketamine in Psychiatric Disorders: Cross-Sectional Survey Study
Source: JMIR Med Educ. 2023 Nov 1;9:e46158. doi: 10.2196/46158 (PMC10652200; doi:10.2196/46158)
Supplement: Multimedia Appendix 2 [file mededu_v9i1e46158_app2.pdf]

Ketamine International Journal Club: Audience Feedback

1-minute survey

Please select the degree to which you agree with the following statements.

OK

1. This is a novel format of online presenting (20min presentation/ 15min chaired Q&A/ 25min informal discussion with attendees 'cameras on').

- ◯ Agree
- ◯ Neither agree nor disagree
- ◯ Disagree

2. This format of presenting is engaging for the speaker and the audience.

- ◯ Agree
- ◯ Neither agree nor disagree
- ◯ Disagree

3. I am satisfied with the time for speaker presentations (20 minutes).

- ◯ Agree
- ◯ Neither agree nor disagree
- ◯ Disagree

4. I am satisfied with the time for the chaired Q&A sessions (15 minutes).

- ◯ Agree
- ◯ Neither agree nor disagree
- ◯ Disagree

5. I am satisfied with the time for informal discussion with attendees (25 minutes).

- ◯ Agree
- ◯ Neither agree nor disagree
- ◯ Disagree

6. I prefer the informal discussion with attendees more than the chaired Q&A sessions.

- ◯ Agree
- ◯ Neither agree nor disagree
- ◯ Disagree

0 of 12 answered

7. The informal discussion with attendees may influence my clinical practice.

- ◯ Agree
- ◯ Neither agree nor disagree

8. The informal discussion with attendees may influence my research.

- ◯ Agree
- ◯ Neither agree nor disagree
- ◯ Disagree

9. I have developed new contacts from the informal discussion with attendees.

- ◯ Agree
- ◯ Neither agree nor disagree
- ◯ Disagree

10. I am satisfied with the quality of the speakers and their presentations.

- ◯ Agree
- ◯ Neither agree nor disagree
- ◯ Disagree

11. Please indicate your primary role:

- ◯ Clinician
- ◯ Student
- ◯ Researcher
- ◯ Psychologist
- ◯ Clinician-Researcher
- ◯ Therapist
- ◯ Other (please specify)

DONE

12. I would recommend this journal club.

- ◯ Agree
- ◯ Neither agree nor disagree
- ◯ Disagree

Please provide any further feedback below (i.e. notifications, speakers, topics, recordings, etc.):

Powered by SurveyMonkey

See how easy it is to create a survey.
