# Supplementary material for: Preliminary Evaluation of a Web-Based International Journal Club for Ketamine in Psychiatric Disorders: Cross-Sectional Survey Study
Source: JMIR Med Educ. 2023 Nov 1;9:e46158. doi: 10.2196/46158 (PMC10652200; doi:10.2196/46158)
Supplement: Multimedia Appendix 3 [file mededu_v9i1e46158_app3.pdf]

Multimedia Appendix 3: Ketamine International Journal Club: Survey Written Feedback

Speaker Comments (3):

1. “Connecting with the journal club and learning from the audience was delightful. The questions asked definitely had me considering novel directions for our team's research. Looking forward to connecting again soon.”
2. “I look forward to this journal club which has developed into a wonderful community! I get more out of this than the usual CME activities that I do.”
3. “It was a pleasure to contribute to the club.”

Audience Comments (8):

1. “I wish for the audience to have a more active role. One example would be; the possibility for the audience to have their Cameras on during the session. Another example would be; during the Q&A sessions, having the possibility to form a queue by raising hands virtually (such as on MS teams) and be allowed to provide Questions in Audio form and possibly video as-well. I believe this would provide a better experience towards full-filling "I have developed new contacts from the informal discussion with attendees" ”.
2. “Interesting topics on TRD as long as you have read the paper in advance”.
3. “Wish that all these are available for review at a later date”.
4. “The only issue I have is that I keep not getting notifications and invites. Not sure why? Thank you”.
5. “Great learning opportunity; as a researcher new to this field, this has been a fantastic forum. Thank you!”
6. “The journal club has been excellent! The balance of clinical and basic science has been perfect, in my humble opinion. The format is very conducive to the combined quality of the speakers and attendees. The hosts are excellent moderators. Regarding statement 3, I endorsed neither agree or disagree because it depends on the topic and speaker. Some need more time to

adequately convey their work (and have often been granted it). And, yes, I have recommended this to many colleagues!”

7. “An evolving field of neurogenesis and its clinical relevance as well as scientific advances.”
8. “I love to participate, love to hear, learn and see those evolving our field further. I cannot think of a better profile of speakers, most of them are researchers and this aggregates a lot to the journal club. I would love to hear some presentations from Dr. James Mourrogh about dextromethorphan, or Dr. Peter Negale about Nitrous Oxide, Dr. Abdallah about ketamine + rapamycin. I would like to hear Dr. McShane's experience with Oxford ketamine clinics as well. I see ASKP as more practical, clinical, day-to-day and journal club more technical.”
